# Dirhodium Tetraacetate Binding to Lysozyme at Body Temperature

**DOI:** 10.3390/ijms26146582

**Published:** 2025-07-09

**Authors:** Gabriella Tito, Giarita Ferraro, Antonello Merlino

**Affiliations:** Department of Chemical Sciences, University of Naples Federico II, 80126 Naples, Italygiarita.ferraro@unina.it (G.F.)

**Keywords:** dirhodium compounds, body temperature crystallography, metal complexes, metallodrugs, protein metalation

## Abstract

Paddlewheel dirhodium complexes are cytotoxic compounds that are also used as catalysts and in the formation of Rh-based artificial metalloenzymes. Low-temperature structures of adducts formed by the model protein hen egg white lysozyme (HEWL) with dirhodium tetraacetate ([Rh_2_(μ-O_2_CCH_3_)_4_]) when crystals of the protein were treated with the metal compound at 20 °C demonstrated that [Rh_2_(μ-O_2_CCH_3_)_4_] in part breaks down upon reaction with HEWL; dimeric Rh-Rh units bind the side chains of Asp18 and the C-terminal carboxylate, and monometallic fragments coordinate the side chains of Arg14 and His15 in 20% ethylene glycol, 0.100 M sodium acetate at pH 4.5 and 0.600 M sodium nitrate, while dimeric Rh-Rh units bind the side chains of Asn93 and Lys96, the C-terminal carboxylate and Asp101, with monometallic fragments that bind the side chains of Lys33 and His15 in 0.010 M HEPES pH 7.5 and 2.00 M sodium formate. To verify whether the binding of this metallodrug to proteins also occurs at body temperature, crystals of HEWL were grown in 0.010 M HEPES pH 7.5 and 2.00 M sodium formate at 37 °C and soaked with [Rh_2_(μ-O_2_CCH_3_)_4_] at the same temperature. X-ray diffraction data collected on these crystals at 37 °C demonstrate that [Rh_2_(μ-O_2_CCH_3_)_4_] reacts with proteins at body temperature. The structures of the Rh/HEWL adduct formed at 20 °C (obtained from data collected at 100 K) and at 37 °C under the same experimental conditions are very similar, with metal binding sites that are conserved. However, metal-containing fragment occupancy is higher in the structure obtained at 37 °C, suggesting a role of temperature in defining the protein metalation process.

## 1. Introduction

X-ray crystallography is routinely used for protein structure determination. The large majority of structures reported in the Protein Data Bank (PDB) have been obtained at 100 K [1], since data collection at this temperature minimizes the formation and diffusion of deleterious reactive species that are formed upon the collision of X-rays with protein atoms and reduces the crystal X-ray diffraction power [2]. Crystallographic experiments at cryogenic temperatures also have other advantages: the low temperature mitigates specific radiation damage [3], allows easy storage and transport of crystals and can be used to trap catalytic intermediates. However, the use of a low temperature is also associated with some disadvantages: functionally relevant enzyme conformations can be overlooked at low temperature; on the other hand, cryogenic temperature structures could show details that are not necessarily significant from a physiological point of view [4]. Recently, there has been growing interest in the search for functionally relevant information that may be missing in low-temperature structures [5]. It has been shown that temperature variations can drive the rearrangement of water molecules bound to a protein structure [6]. Furthermore, physiological temperature can induce conformational changes that hamper the formation of interactions between proteins and metal compounds [7,8]. The body temperature structure of the Nsp3 macrodomain of SARS-CoV-2 revealed a more expanded catalytic site when compared to the structure at low temperature [9], while some local conformational variations were observed in comparing the 310 K structure of SARS-CoV-2 Mpro with that solved at cryogenic and room temperatures [10]. These results have renewed interest in data collection at temperatures higher than 100 K [11].

**Figure 1:**
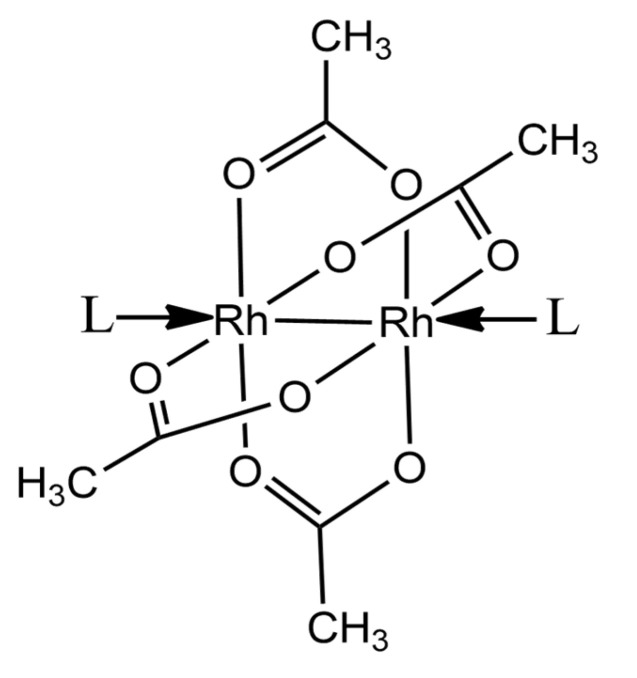
Paddlewheel structure of [Rh_2_(µ-O_2_CCH_3_)_4_]. L indicates possible ligands in the axial position.

Within this framework, very recently, Jacobs, Helliwell and Brink determined the crystal structure of a metal/protein adduct by collecting data at body temperature (37 °C/310 K) [12]. Along the same line, we solved the first structure of a polyoxidovanadate/protein adduct formed at 310 K [13]. A search in the Protein Data Bank revealed that these two X-ray structures are the only ones with reports of detailed atomic models of the interaction between a protein and a metal compound at 310 K. Since structural studies on adducts formed by proteins with metallodrugs at body temperature are important [14,15,16,17], there is a substantial information gap in this field. To obtain further data on this topic, here we studied the interaction of the paddlewheel compound dirhodium tetraacetate ([Rh_2_(μ-O_2_CCH_3_)_4_]) (Figure 1) with the model protein hen egg white lysozyme (HEWL) at body temperature, solving the X-ray structure of the Rh/HEWL adduct formed upon the reaction of the compound with the protein at 310 K. We chose this system since it had already been the subject of structural studies at 100 K [18]. In particular, it has been shown that [Rh_2_(μ-O_2_CCH_3_)_4_], a cytotoxic compound [19,20,21] that is also used as a catalyst [22,23,24,25], in part breaks down upon reaction with HEWL at 20 °C into several metal-containing fragments that are able to bind the protein. HEWL metalation by [Rh_2_(μ-O_2_CCH_3_)_4_] has been studied by solving the crystal structures of Rh/HEWL adducts formed in 20% ethylene glycol, 0.100 M sodium acetate at pH 4.5 and 0.600 M sodium nitrate, and in 0.010 M HEPES pH 7.5 and 2.00 M sodium formate [18]. In the former structure, Rh-Rh units have been found close to the Asp18 side chain and to the C-terminal carboxylate, while monometallic fragments have been found close to the side chains of Arg14 and His15. In the latter structure, Rh-Rh units bind the side chains of Asn93 and Lys96, the C-terminal carboxylate and Asp101, while monometallic fragments are found close to the side chains of Lys33 and His15.

## 2. Results and Discussion

### 2.1. Crystals of the Rh/HEWL Adduct at 310 K

To study HEWL metalation by [Rh_2_(μ-O_2_CCH_3_)_4_], crystals of Rh/HEWL adducts were obtained at 37 °C, treating crystals of the metal-free protein grown at 37 °C in 0.010 M HEPES pH 7.5 and 2.00 M sodium formate with a solution of the reservoir containing a large excess of [Rh_2_(μ-O_2_CCH_3_)_4_] for 1 day. This condition was chosen since it was used to obtain one of the already-solved structures of the Rh/HEWL adduct [18] and allows HEWL crystallization at 37 °C to occur in a short time (1 day). To collect data at 37 °C, it is important to reduce solvent evaporation from the crystal and successive dehydration. This is possible by adding paratone-N oil [26] or closing the protein crystals within a quartz capillary containing a few crystallization drops [12]. The Rh/HEWL adduct crystals were positioned within a borosilicate glass capillary (glass 0500, linear absorption coefficient 71.0 µ cm^−1^) closed with wax, maintained at 37 °C and containing droplets of the crystallization solution stored at 37 °C. X-ray diffraction data at 37 °C were collected at a resolution (2.12 Å, Table 1) that is lower than that of the 100 K structure (1.65 Å). In agreement with previous observations [5], data collected at 37 °C on the crystal of the Rh/HEWL adduct indicate a slight expansion of the cell volume when compared to that of the crystal at 100 K (233789 Å^3^ at 310 K vs. 231255 Å^3^ at 100 K).

### 2.2. Structure of the Rh/HEWL Adduct at 310 K

**Figure 2:**
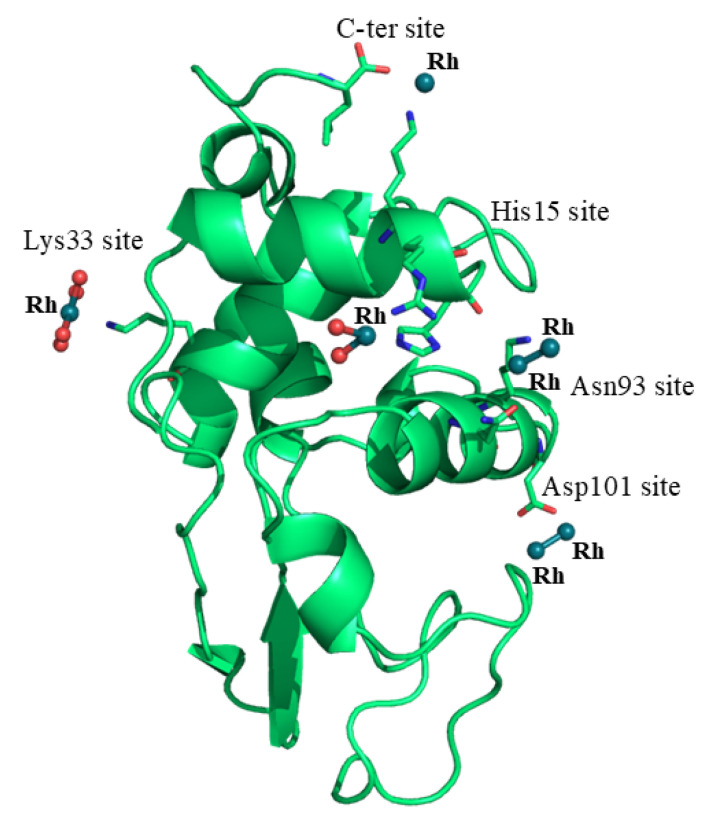
Overall structure of Rh/HEWL adduct at 37 °C. Rh binding sites are evidenced (Rh atoms are colored in dark green, water molecules in red). PDB code: 9RUV.

**Figure 3:**
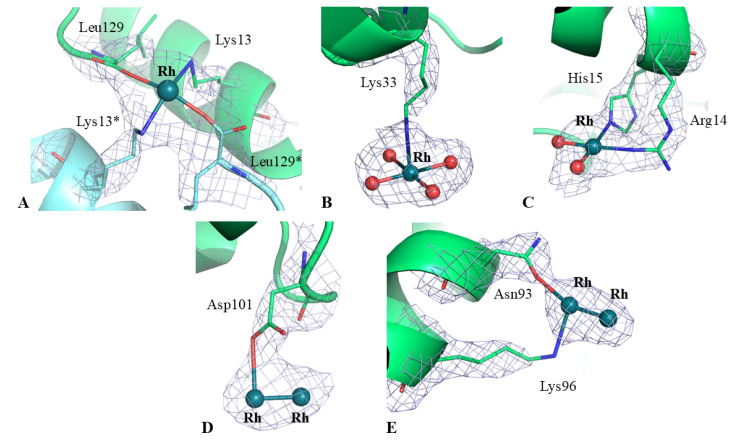
Details of the Rh binding sites in the structure of the Rh/HEWL adduct formed at 37 °C: Lys13 and C-terminal carboxylate (**A**), Lys33 (**B**), Arg14-His15 (**C**), Asp101 (**D**) and Asn93-Lys96 (**E**). 2Fo-Fc electron density maps are contoured at 1.0 σ (light blue). Rh atoms are colored in dark green, water molecules in red. Asterisk (*) indicates residues from symmetry-related molecules.

The structure of the Rh/HEWL adduct at 37 °C contains 129 residues, 24 water molecules interacting with N or O atoms of the protein or water molecules, one sodium ion and seven Rh atoms with six water molecules as metal ligands, and refines with R-factor/R-free values of 0.198/0.261 (Table 1). The structure closely resembles that of the metal-free protein deposited in the PDB under the accession code 193L [27], with a root mean square deviation between the carbon alpha atoms (r.m.s.d.) of 0.30 Å (Figure 2). Inspection of the electron density map revealed the presence of five Rh binding sites (three monometallic centers and two dimetallic centers) on the protein surface. Rh centers are identified close to the C-terminal carboxylate, to the side chain of Lys13 and to the side chains of Lys33, Asn93 and Lys96, Asp101, and Arg14 and His15 (Figure 3).

Close to the side chain of Lys13 and to the C-terminal carboxylate, one Rh atom is observed (occupancy = 0.50). The metal is at a distance of 2.22 Å from the oxygen of the C-terminal carboxylate and 2.50 Å from the Lys nitrogen atom (Figure 3A). Another Rh atom (occupancy = 1.00), which coordinates four water molecules, is found close to the side chain of Lys33 (distance of the metal from the NZ atom of the Lys is 2.39 Å) (Figure 3B). The last monometallic fragment is found close to the side chains of Arg14 and His15 (occupancy = 1.00). At this site, the Rh center coordinates two water molecules, the NE2 atom of the His and the NH2 atom of the Arg, with distances of 2.81 and 2.39 Å, respectively (Figure 3C). Finally, two dirhodium binding sites were identified: the first Rh-Rh center is close to the Asp101 side chain with a distance between the metal and an oxygen of the Asp of 2.90 Å (occupancy = 0.60/0.60). In this region, the Rh–Rh distance is 2.21 Å (Figure 3D). The second Rh-Rh binding site is located close to the side chains of Asn93 and Lys96 (occupancy = 0.50/0.50). Here, one of the Rh atoms of the dirhodium center binds the OD1 atom of Asn93 and NZ atom of Lys96, with distances of 2.44 and 2.65 Å, respectively (Figure 3E). At this dinuclear site, the distance between the two Rh atoms is 2.22 Å.

### 2.3. Structural Comparison with Data from Literature

**Figure 4:**
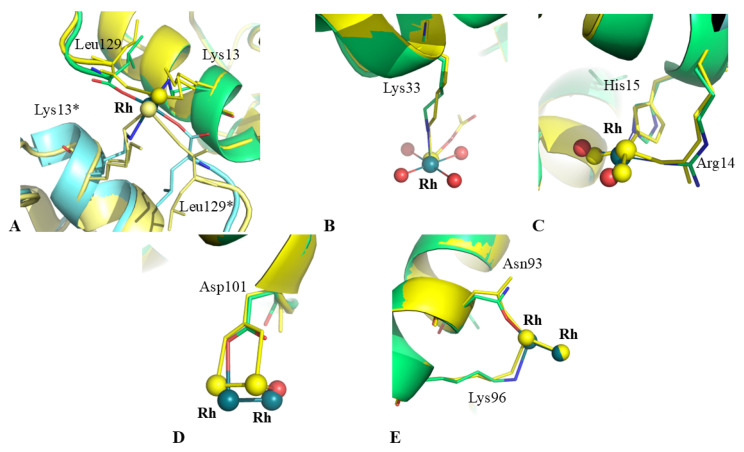
Overlay of the Rh binding sites in the structure at 37 °C vs. that from data collected at 100 K (PDB code 7BE2, yellow). Lys13 and C-terminal carboxylate (**A**), Lys33 (**B**), Arg14-His15 (**C**), Asp101 (**D**) and Asn93-Lys96 (**E**). Rh atoms are colored in dark green, water molecules in red. Asterisk (*) indicates residues from symmetry-related molecules.

X-ray structures of adducts formed upon reaction of [Rh_2_(μ-O_2_CCH_3_)_4_] with HEWL from literature have been obtained by both soaking and cocrystallization procedures [18]. Rh binding sites and metal ligands identified in these structures are reported in Table 2. The overall structure of the Rh/HEWL adduct reported here, deposited in the PDB under the accession code 9RUV, is almost identical to that of the Rh/HEWL adduct formed by soaking procedure under the same experimental conditions at 20 °C and derived from data collected at 100 K (r.m.s.d. = 0.27 Å, PDB code: 7BE2). The two structures show almost the same percentage of residues with hydrogen bonds (86% in the 100 K structure vs. 85% in the structure at 37 °C) and very similar values for the total accessible surface area (6528.8 vs. 6552.0 Å^2^) and for the exposed polar and non-polar accessible surface areas (1754.3 vs. 1695.4 Å^2^ and 3525.5 vs. 3598.2 Å^2^, respectively). Almost all the water molecules constituting the hydration shell of HEWL at 37 °C are conserved in the structure at 100 K. Detailed r.m.s.d. per residue calculations show that the temperature change does not lead to substantial changes in the position of protein atoms. R.m.s. deviations in carbon alpha atom positions in the 37 °C structure from those in the PDB structure with code 7BE2 show that only residues Gly4, 47–49, Pro70, Gly102, Gly117, 121–122 and 126–129 have r.m.s.d. values exceeding 0.40 Å. Notably, residues 47–49, 70, 102, 121–122 and 126–129 are close to Rh binding sites or to Rh binding sites of symmetry-related molecules. The Rh binding sites are also conserved (Table 2), although some modifications in the position of residue side chains involved in Rh binding are observed (Figure 4). This finding indicates that the reactivity of dirhodium tetraacetate with HEWL does not depend on the temperature used for the reaction of the metal complex with this biological macromolecule. This is in agreement with what has been observed in comparing the structure of the HEWL adduct with *fac*-[Et_4_N]_2_[Re(CO)_3_(Br)_3_] and imidazole collected at 100 K [28] and 37 °C [12], while different results have been obtained in comparing the structures of HEWL treated with [V^IV^O(acac)_2_] at 100 and 310 K [13]. A more detailed comparison between the structures of the Rh/HEWL adducts formed at low and body temperatures reveals some differences. In particular, it is interesting to note that the occupancy factors of metal centers in the structure at body temperature are, on average, higher than those found in the structure at 100 K (Table 2), although in the 100 K structure, a longer soaking time was used to obtain the adduct (7 days at 20 °C vs. 1 day at 37 °C). Although caution should be taken in interpreting this result, because of differences in the crystal dimensions and quality, in the diffusion of the metal complex within the crystal and in the soaking time, and possible radiation damage, data suggest that high temperature could enhance the HEWL metalation process by the rhodium compound, in agreement with what was found in the reaction of the same protein with cisplatin [29]. These findings could be significant since they indicate that the number of dirhodium-containing fragments that are bound to proteins at body temperature should be higher than previously believed. This datum also suggests that body temperature should be used to prepare artificial metalloenzymes based on dirhodium compounds since at this temperature, a higher number of Rh-Rh fragments bind proteins.

**Figure 5:**
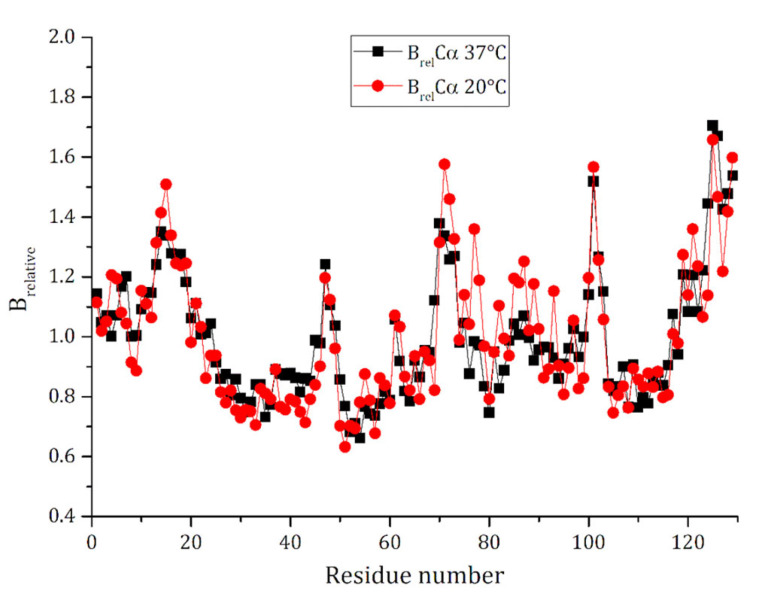
B-relative (B_relative_) versus residue number trend in Rh/HEWL structures obtained at 37 °C and 100 K. B-relative is defined as the ratio between the B-factor of the carbon α atom of each residue and the average B-factor of the carbon α atoms of the whole molecule.

To verify whether the different temperatures used to prepare the crystals of the adduct and to collect X-ray diffraction data and the different occupancies of metal-containing fragments bound to HEWL could influence protein flexibility, a B-factor analysis was carried out. This allowed us to obtain information on the differences in the mobility of protein residues, a feature that is important for protein function. Obviously, B-factors in the 37 °C structure are higher than those found in the 100 K structure. Thus, to carry out a proper comparison, B-relatives (see Materials and Methods for details) were calculated (Figure 5). Inspection of Figure 5 reveals that the B-relative profiles of the two structures are very similar, with 98% of the residues that present differences in B-relatives lower than 0.3.

## 3. Materials and Methods

### 3.1. Crystallization of HEWL and Formation of Rh/HEWL Adduct at 37 °C

[Rh_2_(μ-O_2_CCH_3_)_4_] and HEWL were purchased from Merk Life Science S.r.l. (Milan, Italy) at the highest grade of purity and used without further purification. Crystals of Rh/HEWL adducts reported here were obtained using the soaking strategy at 37 °C. Metal-free HEWL crystals were grown at 37 °C using the sitting drop vapor diffusion method [30]. To obtain these crystals, the protein concentration was increased when compared to the concentration used at 20 °C. In particular, 1 µL of the protein, concentrated up to 100 mg/mL, was mixed with 1 µL of the reservoir solution containing 0.010 M HEPES pH 7.5 and 2.00 M sodium formate. Under these conditions, crystals of the protein grow in less than one day. The protein crystals were then soaked in a solution of the reservoir containing a large excess of [Rh_2_(μ-O_2_CCH_3_)_4_] for 1 day at 37 °C.

### 3.2. Data Collection at 37 °C, Structure Solution and Refinement

To collect X-ray diffraction data at 37 °C, crystals of the Rh/HEWL adduct formed at body temperature using the soaking experiment were fished with a nylon loop and positioned within a glass capillary containing a small drop of the reservoir solution. The capillary was then closed with wax. Data collection was carried out at the XRD2 beamline of ELETTRA synchrotron, Trieste, Italy. A complete data set can be obtained using a crystal orientation of 180° and an oscillation of 1° with an exposure time of 0.05 s per image, allowing a complete data set to be collected within 36 s. Data were scaled using Autoproc [31]. Data collection and refinement statistics are reported in Table 1. The resolution was chosen based on the CC_1/2_ value (cutoff > 0.3). This allowed us to include data at lower signal levels that have been demonstrated to improve protein structure models [32].

Molecular replacement was carried out using Phaser [33] from the CCP4i 7.0 software suite, using PDB code 193L [27] as the search model. Model building was carried out using Coot [34]; refinements were performed with Refmac5 [35].

Geometry and protein statistics were evaluated using the PDB validation server (www.rcsb.org), and structural analysis was carried out using the server VADAR (http://vadar.wishartlab.com/) [36] and Coot routines [34]. To carry out a detailed comparison between the structure of the adduct reported here and that of the same adduct obtained at 100 K, we verified that the radiation damage of the crystal at 37 °C was comparable to that of the low-temperature structure, analyzing the B_damage_ parameter according to Garman and coworkers [37,38].

For a proper comparison of the flexibility of the protein residues, B-relatives were calculated by dividing the B-factor of the carbon alpha atoms of each residue by the average B-factor of the same atoms of the whole protein, as described in reference [39].

## 4. Conclusions

It is known that dirhodium tetracarboxylates bind proteins [20,40,41,42,43,44,45]. Crystals of [Rh_2_(μ-O_2_CCH_3_)_4_] with the model proteins HEWL [18] and bovine pancreatic RNase [46] have been reported; computational studies have been carried out to unveil the molecular bases of the interaction between dirhodium compounds and proteins [47,48]. However, at present, little is known about the effect of temperature on the binding of dirhodium complexes to proteins. Thus, in this work, we have studied the reactivity of dirhodium tetraacetate with HEWL at 37 °C. Our data demonstrate that Rh compounds react with the protein at body temperature, producing results that are very similar to those obtained when soaking was carried out at 20 °C, and by solving the structure of the adduct at 100 K, we found that dirhodium centers and monometallic fragments bind the enzyme at the level of the C-terminal carboxylate and close to the side chains of Lys13, Lys33, Asn93 and Lys96, Asp101, and Arg14 and His15, without altering the proteins overall conformation. These findings suggest that dirhodium compounds could also bind proteins in vivo in the cellular *milieu*. A strict comparison of the structures of the Rh/HEWL adducts obtained under the same experimental conditions using different temperatures for crystal growth and the soaking procedure also suggests that temperature can affect the protein metalation process: the degree of metalation increases at higher temperatures. This result, together with previous data [29], suggests that caution should be taken in administering metallodrugs to patients with fever.

## Figures and Tables

**Table 1:** Data collection and refinement statistics.

Crystal	
Temperature	37 °C
Crystallization condition	0.01 M HEPES pH 7.5 and 2.00 M sodium formate
*Data collection*	
Space group	P4_3_2_1_2
a = b (Å)	80.43
c (Å)	36.14
Resolution range (Å)	56.87–2.12 (2.15–2.12)
Unique reflections	6717 (342)
Completeness (%)	94.0 (100.0)
Redundancy	11.4 (11.8)
Rmerge (%)	0.092 (2.788)
Average I/σ (I)	13.2 (0.9)
CC_1/2_	0.998 (0.405)
Anom. completeness (%)	93.7 (100.0)
Anom. redundancy	6.4 (6.3)
*Refinement*	
Resolution range (Å)	56.87–2.12
N. of reflections	6183
N. of reflections (working set)	382
R-factor/R-free (%)	19.8/26.1
N. of atoms	1043
Average B-factors (Å^2^)	
All atoms	61.2
Rh occupancy	0.50/1.00/1.00/0.60/0.50
Rh atoms	100.5/125.1/159.9/103.2/115.7/134.7/139.1
*R.m.s.d. from ideality*	
Bond lengths (Å)	0.007
Bond angles (°)	1.57
*Ramachandran values (%)*	
Most favored/Additionally allowed	91.9/7.3
Outliers	0.8

**Table 2:** Comparison of Rh binding sites between the structure of the Rh/HEWL adduct obtained at 37 °C and the structure obtained at 20 °C. Table describes the ligands at each Rh binding site. Values in parentheses refer to the occupancy of metal and ligands. Asterisk (*) indicates residues from symmetry-related molecules.

Rh/HEWL Adduct	Crystal at 310 K	Data from Literature	Data from Literature	Data from Literature	Data from Literature	Data from Literature	Data from Literature
**Crystallization condition**	0.01 M HEPES pH 7.5 and 2.0 M sodium formate		0.1 M sodium acetate, 20% ethylene glicol, 0.1 M sodium nitrate				
**PDB code**	9RUV	7BE2	7BDZ	7BE0	7BE1	7BEB	7BEC
**Binding sites**			**Metal fragments at binding sites**				
**C-ter site**	Rh (0.50) Rh * (0.50) Leu129 (1) Leu129 * (1) Lys13 (1) Lys13 * (1)	Rh (0.45) Rh * (0.45) Leu129 (1) Leu129 * (1) Lys13 (1) Lys13 * (1) H_2_O (0.5) H_2_O * (0.5)	Rh (0.65) Rh * (0.65) Leu129 (1) Leu129 * (1) Lys13 (1) Lys13 * (1) Act (0.60) Act (0.60) H_2_O (0.65) H_2_O * (0.65)	Rh (0.65) Rh * (0.65) Leu129 (1) Leu129 * (1) Lys13 (1) Lys13 * (1) Act (0.65) Act (0.65) H_2_O (0.65) H_2_O* (0.65)	Rh (0.65) Rh* (0.65) Leu129 (1) Leu129* (1) Lys13 (1) Lys13* (1) Act (0.65) Act (0.65) H_2_O (0.65) H_2_O * (0.65)	Rh (0.7) Rh * (0.7) Leu129 (1) Leu129 * (1) Lys13 (1) Lys13 * (1) Act (0.7) Act (0.7) H_2_O (0.7) H_2_O * (0.7)	Rh (0.4) Rh * (0.4) Leu129 (1) Leu129 * (1) Lys13 (1) Lys13 * (1)
**Lys33 site**	Rh (1) Lys33 (1) H_2_O (1) H_2_O (1) H_2_O (1) H_2_O (1)	Rh (0.20) Lys33 (1) Act (0.33)	-	-	-	-	-
**His15 site**	Rh (1) His15 (1) Arg14 (1) H_2_O (1) H_2_O (1)	Rh (0.35) His15 (1) Arg14 (1) H_2_O (0.35) H_2_O (0.35)	Rh (0.35) His15 (1) Arg14 (1) Act (1) H_2_O (0.35) H_2_O (0.65)	Rh (0.5) His15 (1) Arg14 (1) Act (1) H_2_O (0.5)	Rh (0.3) His15 (1) Arg14 (0.5) Act (1)	Rh (0.3) His15 (1) Arg14 (0.5) Act (1) H_2_O (0.3) H_2_O (0.3)	Rh (0.25) His15 (1) Arg14 (0.5) Act (1) H_2_O (1)
**Asp101 site**	Rh (0.60) Rh (0.60) Asp101 (1)	Rh (0.40) Rh (0.40) Asp101 (1) H_2_O (0.40)	-	-	-	-	-
**Asn93 site**	Rh (0.50) Rh (0.50) Asn93 (1) Lys96 (1)	Rh (0.30) Rh (0.30) Asn93 (1) Lys96 (1) H_2_O (1)	-	-	-	-	-
**Asp18 site**	-	-	Rh (0.40) Rh (0.40) Asp18 (1)	Rh (0.20) Rh (0.20) Asp18 (1) H_2_O (0.20)	-	-	-

## Data Availability

Data is contained within the article.

## Abbreviations

CD	Circular dichroism
HEWL	Hen egg white lysozyme
PDB	Protein Data Bank
R.m.s.d.	Root mean square deviation
RNase A	Bovine pancreatic ribonuclease
